# Supplementary material for: The prevalence of mental illness in refugees and asylum seekers: A systematic review and meta-analysis
Source: PLoS Med. 2020 Sep 21;17(9):e1003337. doi: 10.1371/journal.pmed.1003337 (PMC7505461; doi:10.1371/journal.pmed.1003337)
Supplement: S1 Table — MeSH term, Medical Subject Headings. (DOCX) [file pmed.1003337.s002.docx]

**S1 Table – Summary of terms used in the Medline search strategy**

| 1 | Refugees [MeSH term] |
| --- | --- |
| 2 | refugee* [text word] |
| 3 | asylum-seek*[text word] |
| 4 | Or/1-3 |
| 5 | Mental Health [MeSH term] |
| 6 | mental*[text word] |
| 7 | Mental Disorders [MeSH term] |
| 8 | mental disorder*[text word] |
| 9 | mental illness* [text word] |
| 10 | Depressive Disorder/di [diagnosis] [MeSh term] |
| 11 | depress*[text word] |
| 12 | Anxiety Disorders/di [diagnosis] [MeSH term] |
| 13 | anxiety*[text word] |
| 14 | Agoraphobia/di [diagnosis] [MeSH term] |
| 15 | agoraphobi* [text word] |
| 16 | Phobic Disorders/di [diagnosis] [MeSH term] |
| 17 | phobi*[text word] |
| 18 | social anxi*[text word] |
| 19 | Trauma [text word] |
| 20 | Stress Disorders, Post-Traumatic/di [diagnosis] [MeSH term] |
| 21 | PTSD* [text word] |
| 22 | post?traumatic stress disorder*[text word] |
| 23 | Psychotic Disorders/di [diagnosis] [MeSH term] |
| 24 | psych* [text word] |
| 25 | Schizophrenia/di [Diagnosis] [MeSH term] |
| 26 | schizo* [text word] |
| 27 | Bipolar Disorder/di [Diagnosis] [MeSH term] |
| 28 | bipolar* [text word] |
| 29 | Torture [MeSH term] |
| 30 | torture* [text word] |
| 31 | emotional disorder* [text word] |
| 32 | emotional disturbance* [text word] |
| 33 | Autism Spectrum Disorder/di [Diagnosis] [MeSH term] |
| 34 | autism Spectrum Disorder [text word] |
| 35 | autis* [text word] |
| 36 | Attention Deficit Disorder with Hyperactivity/di [Diagnosis] [MeSH term] |
| 37 | attention deficit disorder [text word] |
| 38 | ADHD [text word] |
| 39 | Or/5-38 |

Abbreviations: MeSH term = Medical Subject Headings, * = truncation symbol
