# Supplementary material for: The prevalence of mental illness in refugees and asylum seekers: A systematic review and meta-analysis
Source: PLoS Med. 2020 Sep 21;17(9):e1003337. doi: 10.1371/journal.pmed.1003337 (PMC7505461; doi:10.1371/journal.pmed.1003337)
Supplement: S2 Egger’s Test Depression — Figure: Funnel plot using data from 17 studies providing data for the prevalence of depression. Each dot represents a study. ES, effect size; s.e, standard error. Table: Egger’s test set at a threshold of a p-value less than 0.05 to indicate funnel plot asymmetry. Coef., coefficient; Conf. Interval, confidence interval; Std_Eff, standard effect; Std. Err, standard error; Test of HO, test of null hypothesis. (DOCX) [file pmed.1003337.s005.docx]

Egger’s test plot depression

**S2 Fig.** Funnel plot using data from 17 studies providing data for the prevalence of depression. Each dot represents a study. ES = effect size, s.e = standard error.

**S2 Table**. Egger’s test set at a threshold of a *p* value less than 0.05 to indicate funnel plot asymmetry. Std_Eff = standard effect; Coef. = coefficient; Std. Err = Standard Error; Conf. Interval = confidence interval; Test of HO = test of null hypothesis.
