# Supplementary material for: The prevalence of mental illness in refugees and asylum seekers: A systematic review and meta-analysis
Source: PLoS Med. 2020 Sep 21;17(9):e1003337. doi: 10.1371/journal.pmed.1003337 (PMC7505461; doi:10.1371/journal.pmed.1003337)
Supplement: Attachment — Submitted filename: Response to reviewers_PLOS Medicine vers 3.pdf [file pmed.1003337.s008.pdf]

Response to Reviewers' Comments

Requests from the editors:

1.Abstract: Please structure your abstract using the PLOS Medicine headings (Background, Methods and Findings, Conclusions). Please report your abstract according to PRISMA for abstracts, http://www.plosmedicine.org/article/info:doi/10.1371/journal.pmed.1001419 .
Author Response: *We have consulted the PRISMA guidelines for abstracts and revised accordingly. Please see the changes to the abstract highlighted in the text.*

2. Abstract: Background (second sentence): Please revise to "...has reported wide variation in mental illness prevalence data...."
Author Response: *This has been revised and the changes have been highlighted in the abstract.*

3. Abstract: Methods and Findings: Please provide the data sources, types of study designs included, eligibility criteria, and the synthesis and appraisal methods.
Author Response: *The Methods and Findings section of the Abstract has been revised to provide further details regarding data sources, types of studies included, eligibility criteria, synthesis, and appraisal methods. The changes have been highlighted in the Abstract:*

"A comprehensive search of electronic databases was undertaken from 1 January 2003 to 4 February 2020 (MEDLINE, MEDLINE In-Process, EBM Reviews, Embase, PsycINFO, CINAHL, PILOTS, Web of Science). Quantitative studies were included if diagnosis of mental illness involved a clinical interview and use of a validated assessment measure, and reported at least 50 participants. Study quality was assessed using a descriptive approach based on a template according to study design (modified Newcastle-Ottawa Scale). Random effects, based on inverse variance weights, were conducted. Subgroup analyses were performed for sex, sample size, displacement duration, visa status, country of origin, and type of residence. The systematic review was registered with PROSPERO (CRD) 42016046349).

The search yielded a result of 21,842 records. Twenty-six studies, which included one randomized controlled trial and 25 observational studies, provided results for 5,143 adult refugees and asylum seekers. The prevalence of post-traumatic stress disorder (PTSD) was 31.46% (95% CI 24.43-38.5), depression was 31.5% (95% CI 22.6-40.38), anxiety disorders were 11% (95% CI 6.75-15.43), and psychosis was 1.51% (95% CI 0·63-2·40). Substantial heterogeneity was present in the prevalence estimates of PTSD, depression, and anxiety, and limited covariates were reported in the included studies."

4. Abstract: Methods and Findings: In the last sentence of the Abstract Methods and Findings section, please describe the main limitation(s) of the study's methodology.

Author Response: *A sentence has been added to the end of the Methods and Findings section of the Abstract which describes the main limitation of the review's methodology. It now states the following:*

"Substantial heterogeneity occurred in the prevalence estimates of PTSD, depression, and anxiety, and limited covariates were reported in the included studies.

5. Author Summary: At this stage, we ask that you include a short, non-technical Author Summary of your research to make findings accessible to a wide audience that includes both scientists and non-scientists. The Author Summary should immediately follow the Abstract in your revised manuscript. This text is subject to editorial change and should be distinct from the scientific abstract. Please see our author guidelines for more information: https://journals.plos.org/plosmedicine/s/revising-your-manuscript#loc-author-summary

Author Response: *We have provided a brief, non-technical author summary which has now been included immediately following the Abstract.*

6. Methods: Please update your search to the present time. We require that SRs are updated to within roughly six months of the expected publication date. Your search has not been re-run since February of 2018.

Author Response: *The search has now been updated to 4 February 2020. This resulted in the inclusion of five further studies published between 2018 and 2019, bringing the total to 26 included studies and providing data for 5,143 adult refugees and asylum seekers. These studies provided additional data for the outcomes of PTSD, depression, anxiety, and psychosis and for the subgroup analyses.*

7. Methods (Lines 144-145): Please describe the evaluation of study quality. Specifically, it is stated in the methods that: "Individual items related to study quality such as internal and external validity, reporting bias, and conflict of interest were assessed." Please describe how these items were analyzed, and how this assessment factored into the results of the review and meta-analysis.

Author Response: *Thank you for this feedback. We have now added more details regarding the process and evaluation of study quality. We have described which additional components of risk of bias were analysed as part of the template used, to extend the Newcastle-Ottawa Scale (NOS). At the end of the Results section, we have provided a summary of the outcomes of the risk of bias assessments. This includes the overall ratings, reasons for ratings, and a discussion of key papers and their influence on the prevalence estimates.*

Please see the following changes highlighted in the Method section underneath the heading Risk of Bias:

"Methodological quality was independently assessed by two reviewers (RB and JAB) using a risk of bias assessment template developed a priori according to study design, in which the criteria to assess a randomized controlled trial (RCT) differs to the criteria of an observational study.[17] These templates are based upon the Newcastle-Ottawa Scale (NOS)[18], with the addition of further risk of bias components assessing internal and external validity such as use of appropriate study design, explicit and appropriate use of inclusion criteria, reporting bias, confounding, sufficient power for analyses, and any apparent conflicts of

interest. These modified and extended templates have been used in international evidence-based guidelines and other systematic reviews.[19-21] *Using a descriptive approach, studies were assigned a rating of low, moderate, or high risk of bias. Any disagreement was resolved by discussion with other reviewers (MGH and MF) to reach a consensus. Such discussions occurred on two occasions, both times regarding papers assigned a high risk of bias.* [22,23] *"*

8. Methods (Lines 128-130): Please describe the random effects model used in the meta-analysis.

Author Response: *We have added further details to the Methods section to describe the random effects model used in this meta-analysis. The changes have been highlighted in the Methods section commencing from line 171:*

"Random effects meta-analyses using a DerSimonian and Laird estimator based on inverse variance weights were employed. [20] *Random effects meta-analysis was chosen as heterogeneity was anticipated because of between-study variations in clinical factors due to the heterogenous nature of refugees and asylum seekers (e.g. country of origin, language, host nations etc.). The DerSimonian and Laird method incorporates a measure of the heterogeneity between studies. Heterogeneity was assessed using the* I^2 *statistic.* [21]*"*

9. Methods: Please remove the section titled: "Role of the Funding Source" as this information is extracted from the manuscript submission system automatically.

Author Response: *This has been removed and the change has been highlighted in the manuscript.*

10. Results: Please provide 95% CIs and p values for the results discussed for the subgroup analyses for each mental illness described (e.g. PTSD paragraph, depression paragraph, etc.).

Author Response: *We have updated the results section to include the 95% confidence intervals and p values for the subgroup analyses for each mental illness. The changes have*

been highlighted in the Results section.

11. Results: Please provide numerators and denominators for overall prevalence rates, if not in the text then at a minimum present these in the appropriate tables.

Author Response: *We have updated the results section to include the numerators and denominators for the overall prevalence rates for each mental illness. The changes have been highlighted in the text. We do have some concerns that this information may be misleading and contribute to confusion because the calculation of the overall pooled prevalence involved an inverse weighting method and was not simply the direct summation of reported prevalence across studies.*

12. Discussion: Please expand on your Discussion as follows: Please increase the discussion of the existing research on prevalence of mental illnesses in the refugee and asylum-seeking population, extending the depth of your discussion beyond the 2005 Fazel et al. and 2009 Steel et al. reviews if possible. Please expand on your discussion of subgroup analysis findings regarding why PTSD prevalence may be higher in individuals originating from Africa.

Author Response: *Thank you for this feedback. As a result, we have revised the discussion in order to offer the reader more depth and insight into the results from this systematic review. We have now included two additional studies for comparison, extended the discussion regarding the subgroup analysis results, as well as expanding upon the initial discussion of the literature both current and historical in the Introduction (please see paragraph 2 of the Introduction).*

Please see the following paragraphs in the Discussion, changes have been highlighted.
Line 341:

"The studies with populations from Africa reported the highest prevalence of PTSD. This result likely reflects how countries within Africa are consistently ranked at the highest levels of the Political Terror Scale. [69] This scale is a five-point rating system based on data from Amnesty International and the U.S State Department and measures the levels of extensive human rights violations and violence within nations…"

Line 357:

"The prevalence of PTSD and depression is higher than in the review by Fazel et al. [6] This could reflect the fact that this current systematic review included refugee populations from low- and middle-income countries or that the more recent refugee flows might be exposed to higher numbers of risk factors. The results for anxiety disorders and psychosis are comparable. The influence of sample size is further supported, with the larger studies reporting lower prevalence rates for PTSD and depression. However, this was not the case for anxiety where sample size did not influence prevalence. The results for PTSD and depression are comparable to the findings of Steel et al. [7] and slightly lower than other systematic reviews which have reported PTSD prevalence in the range of 36% - 43% and depression 40% - 44%. [12,73] "

13. Discussion (Line 144-145, second to last paragraph): Please avoid assertions of primacy and add "To the best of our knowledge…" or similar to the following sentence: "It is also the first systematic review to place no restrictions on language or on countries of origin or settlement."

Author Response: *Thank you for this feedback. We have made the following edits to this sentence in the discussions. Please see the changes highlighted in the text:*

"To the best of our knowledge, the is the first systematic review to place no restrictions on language or on countries of origin or settlement."

14. Figures 2, 4, 6, 8, and 9: Please describe in the figure legend the meaning of the vertical dashed red line.

Author Response: *We have added the following description to the above-mentioned figures: "The dashed red line shows the position of the overall prevalence."*

15. Figures 2-9: Please provide an X-axis label for these graphs.

Author Response: *We have added the title "Prevalence %" to the x-axis for all the figures.*

16. References: Please use brackets for in-text reference numbers, e.g. [1].

Author Response: *We have made this change in formatting the reference numbers. In order to maintain readability of the document, these changes were not tracked.*

17. Supporting Information: Please provide separate labels and titles (e.g. S1 Text, S1 Table, S1 Figure), and legends for all figures and tables. This includes the material included in your Appendix (Checklist, example search string, bias assessment template, and the three Egger plots for PTSD, Depression, and Anxiety). Please refer to our guidelines at: https://journals.plos.org/plosmedicine/s/submission-guidelines#loc-supporting-information

Author Response: *We have consulted the guidelines and have provided figure captions, including the legends in the manuscript. The figures captions appear in read order, immediately following the paragraph where each figure is first cited. Separate labels for the supporting information are now included in the manuscript and have been cited appropriately in the body of the manuscript.*

18. Supporting information: Please define all abbreviations used within the figures and tables in the accompanying legends.

Author Response: *A list of abbreviations has been included on the title page of the manuscript. We have limited the use of abbreviations in the figures. The abbreviations for table 1 are listed in the manuscript immediately following the paragraph where it has been cited for the first time.*

19. Supporting information: In your cover letter, you mentioned that the findings related to children and adolescents have been written up separately and that report is under consideration at another journal. Please include a copy of the unpublished manuscript as part of the supporting information with your revised submission, and also include a paragraph in your cover letter describing the key differences and any overlap between the two papers.

Author Response: *We have included a copy of the (now published) child and adolescent systematic review manuscript with the submission. We have also added further details to the cover letter to outline the overlap and differences between these two reviews.*

20. Checklist: Thank you for providing the PRISMA checklist. Instead of page numbers, please use sections and paragraphs when referring to locations within the article.
Author Response: *We have updated the provided PRISMA checklist to reference sections and paragraphs instead of page number.*

Comments from the reviewers:

Reviewer #1: Thank for the opportunity to review this paper. Blackmore and colleagues report findings from a systematic review which synthesizes mostly survey-based studies to describe the prevalence of mental illness in adult refugees and asylum seekers, reporting pooled results in post-traumatic stress disorder, depression and anxiety. All prevalence figures were higher in refugees than in the general population, which in itself not surprising. This paper adds updated figures that are more recent compared to some previous reviews done completed previously. I do, however, have some suggestions/comments primarily on the methodology.

Abstract
In general, the abstract needs a bit more detail when reporting the methods and findings, in particular:

Lines 38-39: What "strict" inclusion criteria was described here? This is presented in the main text but the abstract should also explicitly state this directly.
Author Response: *Thank you for this feedback. We have revised this sentence in the Abstract to be more explicit in the details of the strict inclusion criteria.*
The sentence now reads:

"Quantitative studies were included if diagnosis of mental illness involved a clinical interview and use of a validated assessment measure, and reported at least 50 participants."

Lines 40-41: Study quality assessed should be stated/assessed by the specific tool used. Abstract methods in general: Methods don't specify what type of meta-analyses was utilised? Random-effects and type of weighting method should be stated. Specify also this was combining aggregate prevalence measures reported in each study.

Author Response: *We have revised the Methods and Findings section of the Abstract. Details have been added to further describe the process undertaken to complete the quality assessment of included studies. Please see the following changes to the Abstract, line 47:*

> *"Study quality was assessed using a descriptive approach based on a template according to study design (modified Newcastle-Ottawa Scale)."*

We have added the following details to the Abstract regarding the random-effects model used in this meta-analysis, please see line 48:

> *"Random effects, based on inverse variance weights, were conducted."*

We have removed the sentence in the Method section which stated that prevalence rates of mental illnesses were combined by direct summation of numerators and denominators across studies as this was misleading.

Line 44: This jumps out without any context - meta-analyses performed in just larger studies, then this needs to pre-specified in the methods section of the abstract or it just looks like selective reporting of a particular sub-analyses.

Author Response: *The details of all of the planned subgroup analyses have now been listed in the Abstract. Please see the following changes starting from line 49 of the Abstract:*

> *"Subgroup analyses were performed for sex, sample size, displacement duration, visa status, country of origin, and type of residence"*

Methods

Firstly, my main concern on the methods is that as stated in Lines 93-96, the search strategy was not entirely the same as the earlier review performed by Fazel et al., and thus I question whether the rationale to limit the date to 1 Jan 2003 onwards appropriate. Because the search strategy is in fact different in this current review, it is not a "pure" an update on the Fazel et al

review. The expanded search, with different criteria, pre-2003 may in fact pick up slightly different composition of studies from Fazel et al. because the authors imposed stricter criteria on mental health diagnoses and expanded the range of databases searched, number of search terms, and no restriction on geography or language.
Second, even if the rationale was purely to update the Fazel et al. review, using the exact search strategy, any evidence synthesis and meta-analyses performed should also include the studies identified in the previous date for the update. Normally, Cochrane Review updates would in fact include both the previous studies combined with new studies. Here, the focus is only on new studies, but I'm not sure this is entirely rationale approach or at least has not been rationalised strongly enough.

Author Response: *We have reviewed each section of the manuscript and revised it where necessary to clarify that this review is based on the methods of Fazel et al. but is not a pure update. We believe that an estimate based on current refugee populations would have more real-world relevance and so we decided to focus on relatively recent studies. Additionally, the standards for research reporting have changed considerably over the past two decades (such as details of the diagnostic assessment methods used and the covariates expected to be reported), and the quantity of research in this field has also vastly increased (and availability of publications other than in English or from countries of transition or first resettlement). These changes contributed to our decisions regarding this review's scope and search strategy.*

We have added the following sentences to the Methods section, please see the following changes starting from line 131:

"The search was based on that used in the earlier systematic review of Fazel et al. [6] but expanded to increase the range of databases searched, number of search terms, and stricter criteria regarding study inclusion…"

Line 137:

"This start date reflects the end date of the search conducted by Fazel et al. [6], in order to provide a contemporary estimate of mental illness within this population."

Line 103: Clarify if there were any restrictions on study design: i.e. cohort, case-control, cross-sectional surveys (though it looks like most studies were surveys)

Author Response: *A sentence has been added to the Method section to clarify the restrictions placed on study design. Please see line 151 in the Methods section for the highlighted changes:*

"Randomized controlled trials, longitudinal cohort, and cross-sectional studies were considered for inclusion whereas retrospective registry reviews, medical records audits and qualitative studies were excluded. Case-control studies were excluded if cases were selected based on the presence of our outcomes of interest."

Lines 129-130: What weighting methods was utilised in the random-effects model
Author Response: *We have provided information regarding the weighting method used in this systematic review. The following sentence has been added to the Abstract, line 48:*

"Random effects, based on inverse variance weights, were conducted."

The following sentences have been added to the Methods section to provide further information regarding the random effects model and weighting method used in this systematic review. Please see line 172:

"Random effects meta-analyses using a DerSimonian and Laird estimator based on inverse variance weights were employed. [20] *Random effects meta-analysis was chosen as heterogeneity was anticipated because of between-study variations in clinical factors due to the heterogenous nature of refugees and asylum seekers (e.g. country of origin, language, host nations etc.). The DerSimonian and Laird method incorporates a measure of the heterogeneity between studies. Heterogeneity was assessed using the I2 statistic.* [21]*"*

Lines 134-135: This is the primary concern with the weighting methods. The authors state earlier they used a random-effects model to account for heterogeneity but then describe here that "prevalence rates were combined by direction summation of numerators and denominators". This would suggest that the prevalence rates were simply combined by direct summation across studies, which of course does not account for unequal weighting given from various studies due to sample size and heterogeneity in study design. The definition of prevalence implies a standard statistical assumption following a binomial distribution. Hence, the pooling of prevalence needs to consider the variance derived from the binomial

distribution, accounting for the size of the study: var(p) = p(1-p)/N, p is the prevalence and N is the population size

Then the pooled prevalence can be combined using the inverse variance method and the model should be specified with a random-effects term to account for heterogeneity. 95% CI can be appropriately be computed using either the exact method, score method, Wald's method. see for the methodology: https://jech.bmj.com/content/67/11/974

It can be implemented in STATA using metaprop command: see https://archpublichealth.biomedcentral.com/articles/10.1186/2049-3258-72-39

Author Response: *Thank you for this feedback. We have* ***removed*** *the following sentence from the Methods section to improve clarity.*

"Prevalence rates of mental illnesses were combined by direct summation of numerators and denominators across studies, thereby providing a pooled estimate."

The methodology suggested, inverse variance weighting, is in fact the methodology applied in the analysis of this systematic review. We have revised the Abstract and Methods section to now include more precise details regarding the random effects model and weighting methods that we used. These changes have been highlighted in the text.

Please see line 171 of the Method section for the following addition:

"Meta-analysis results (Stata software version 14.1 (StataCorp LP)) were expressed as prevalence estimates of mental illness calculated with 95% confidence intervals (CIs) in the pooled data. Random effects meta-analyses using a DerSimonian and Laird estimator based on inverse variance weights were employed. [20] *Random effects meta-analysis was chosen as heterogeneity was anticipated because of between-study variations in clinical factors due to the heterogenous nature of refugees and asylum seekers (e.g. country of origin, language, host nations etc.). The DerSimonian and Laird method incorporates a measure of the heterogeneity between studies. Heterogeneity was assessed using the I2 statistic.* [21]*"*

Lines 138-139: Limiting studies stratified by participant number: This shouldn't be necessary with proper weighting methods when pooling (such as Inverse-variance or DerSimonian and Laird method), as small study effects will have large 95% CI and contribute fairly small effects to the overall pooled results. Doing this arbitrary stratification in the primary analyses actually introduces some bias itself.

Author Response: *The decision to report the influence of sample size on mental illness prevalence was guided by the earlier systematic review by Fazel et al. 2005, who observed that the prevalence rates for the larger studies (n>200) were significantly lower than those reported in the smaller studies. We felt that the readers would be interested in the replication of a similar investigation and as a result listed it as one of our variables of interest in our subgroup analyses. After consulting with our statistician, we can confirm that this subgroup analysis is in fact a different analysis, as compared to the results obtained from the meta-analysis and study weighting procedures. It does offer informative results, particularly when compared to the earlier findings and as such we would like to keep this analysis included.*

Lines 142-143: The NOS scale is useful but really designed for Cohort and Case-control studies, hence most of the questions refer to selection bias, control for confounding, and selection of comparators, which all cross-sectional studies are not designed to capture. AXIS-tool is more relevant for assessing quality of cross-sectional studies https://bmjopen.bmj.com/content/6/12/e011458

Author Response: *The risk of bias appraisal tool used is based upon the Newcastle Ottawa Scale (NOS), but has been modified to include additional components to further assess internal and external study validity. Additionally, as part of the appraisal process different templates are used for each type of study design. Therefore, the template we used for the observational studies was different to that used for the appraisal of the randomised controlled trial. The risk of bias paragraph in the Method section has been updated to provide further details regarding this appraisal tool and process. Please see the changes below, starting from line 186:*

"Methodological quality was independently assessed by two reviewers (RB and JAB) using a risk of bias assessment template developed a priori according to study design, which

meant the criteria to assess a randomized controlled trial (RCT) was different to the criteria of an observational study (S3).[26] These templates are based upon the Newcastle-Ottawa Scale (NOS)[27], with the addition of further risk of bias components assessing internal and external validity such as use of appropriate study design, explicit and appropriate use of inclusion criteria, reporting bias, confounding, sufficient power for analyses, and any apparent conflicts of interest; as has been used in international evidence-based guidelines and other systematic reviews.[28-30] Using a descriptive approach, studies were assigned a rating of low, moderate, or high risk of bias. Any disagreement was resolved by discussion with other reviewers (MGH and MF) to reach a consensus..."

Reviewer #2:

Thank you for your work on this systematic review and meta-analysis on the Prevalence of Mental Illness in Refugees and Asylum Seekers.

Strengths of your manuscript include:
- expanding the evidence base on psychosis among refugees and asylum seekers
- This work continues to demonstrate that refugees experience long term psychiatric sequelae of traumatic events experienced as a result of refugee status and as you note call for long-term health care beyond the initial period of resettlement- very important to ensure funding and programmatic planning.

I am suggesting several points that should be addressed prior to publication that can improve the utility of this work:

- My main concern is that although it is noted that there is some novel information in this review, besides the interesting information on psychotic symptoms, this review essentially replicates what we know from the literature on PTSD for the past 20 years. The problem is a political one that translates to underfunded programs and poor to no policies based on the evidence base, globally. The majority of refugees and asylum seekers do not live-in high-

income countries- they face significant challenges in settings where there is little to no mental health care. As such, it would be good if you could address, even very briefly, the broader context for having up to date estimates of poor mental health among refugees and asylum seekers globally. Chronic PTSD prevents integration into new societies and reintegration. Funding such programs now makes good economic sense as well as having a human rights imperative.

Author Response: *Thank you for this feedback. The authors agree that these are important points to make in this paper. We have expanded upon both the Introduction and the Discussion sections to better demonstrate and highlight the new information this review contributes, as well as the value and progression it adds to the field of refugee mental health.*

Introduction, line 103:

"Not only is there is a lack of research which includes all global refugee populations, there is also a lack of research investigating the full breadth of mental illness as the literature has mainly focused on PTSD and depression."

Introduction, line 113:

"Current prevalence information could be a powerful tool for advocacy and also assist host countries and humanitarian agencies to strengthen health services to provide the essential components of timely diagnosis and treatment for mental illnesses, in line with the priorities and objectives of the World Health Organization's Draft Global Action Plan 'Promoting the health of refugees and migrants' (2019-2023).[14] Providing appropriate, early, and ongoing mental health care to refugees and asylum seekers benefits not only the individual but the host nation, as it improves the chances of successful reintegration which has long-term benefits for the social and economic capital of that country, not only for the displaced generation but will likely impact on the second generation as well. [15] Bringing together the global literature on the prevalence of mental illness in refugee and asylum seeker populations would also enable the research community to move ahead and focus on different components of the mental health

needs of this population, for example on interventions, on less well understood mental health conditions or longitudinal mental health trajectories."

Discussion, line 321:

"Despite this high heterogeneity, which is expected when investigating and analyzing prevalence across global refugee populations, knowledge of current prevalence estimates provides a foundation for the field to build on. Researchers can progress with this knowledge and focus their attention on addressing the critical need for immediate, appropriate, and ongoing mental health support and interventions. Without the progression of further high-quality research which explores the different components of mental health needs, culturally appropriate and effective interventions, and longitudinal mental illness trajectories, untreated mental illnesses will severely impact upon successful integration into host communities. For host countries and humanitarian agencies current prevalence estimates of mental illness within this ever-growing population can be used in advocacy and health service planning to strengthen mental health services for refugees and asylum seekers, in line with WHO priorities and objectives. [14]"

- It is discussed that it's a strength that results from medical settings were included (excluding survey results) but I'm not sure that's a strength. While in some settings, it can be "assumed" that this means that a diagnosis of say, PTSD, is likely more accurate, it still depends on the clinician, their level of training, need for a quick diagnosis etc etc. It also has a potential country setting bias- biased towards places that may have higher capacity to diagnose and treat PTSD. This should be clearly stated.

Author Response: *The authors agree that including results from medical settings can result in some bias. To address this, we excluded any studies that recruited participants from psychiatric or mental health clinics, but we included studies that recruited individuals from general refugee health or primary health care clinics. We also excluded studies that relied on*

case reports such as medical records audits or analysis of administrative data from health services, for the reasons that reviewer highlights. We have clarified these points in line 152 and 156 of the Methods section.

- It's concerning that there is such heterogeneity among study results, even though the authors admirably sought to address this. It could be due to your review's criteria and that the results are drawn from such different settings and populations- these populations have experienced such different experiences and culturally may manage them differently. The authors state that this means that "The results of the meta-analysis yielded high statistical heterogeneity, which is evidence of the critical need for research in this field that is large-scale, uses rigorous diagnostic methods, and characterizes the study sample in detail." but this is highly unlikely as the reason, given the 20 years of replicable results already clear in the literature. Again, this is likely the result of the review's criteria.

Author Response: *Thank you for this feedback. Few restrictions were placed on characteristics of the refugee experience, in the hope of including all possible studies and ascertaining a global prevalence estimate. As a result, the meta-analysis yielded expectedly high statistical heterogeneity. However, despite this high heterogeneity, the results provide an important update on the estimated prevalence of mental illness in the global refugee community. We have added some sentences to the discussion to highlight this, starting at line 320:*

"With the aim of including all possible studies, this systematic review placed few restrictions on the characteristics of the refugee experience and as a result, the meta-analysis yielded substantial statistical heterogeneity. Despite this high heterogeneity, which is expected when investigating and analyzing prevalence across global refugee populations, knowledge of current prevalence estimates provides a foundation for the field to build on. Researchers can progress with this knowledge and focus their attention on addressing the critical need for immediate, appropriate, and ongoing mental health support and interventions."

- The lower anxiety results is puzzling. PTSD and anxiety disorders are highly

comorbid. More explanation needs to be given for this including whether the review's strategy led to potentially inaccurate results.

Author Response: *Thank you for this feedback. We have expanded upon this finding in the Discussion section. Please see the following additions, line 314:*

"Only eleven studies reporting data on anxiety prevalence met the inclusion criteria for this review, and of those eleven only six assessed the full range of DSM anxiety disorders. With a heavy emphasis on PTSD and depression, the full breadth of anxiety disorders is less frequently examined and reported in the literature. It was only recently, with the release of DSM 5, that PTSD was no longer classified as an anxiety disorder but in a separate category of trauma and stressor related disorders. [60] *Further research on the prevalence of the full range of anxiety disorders and comorbidities is needed."*

Reviewer #3:

The authors have undertaken a systematic review and meta-analysis of epidemiological studies reporting the prevalence of mental disorder amongst refugee and asylum seekers. The review reported is very closely modelled on an earlier review undertaken by Fazel et al (2005). As with that review the authors have restricted their review to research studies that have used structured or semi-structured diagnostic instruments and have excluded studies that report prevalence estimates derived from screening or self-report measures.

The prevalence estimates cited on page 3, line 72-74 are not so relevant to the current review given the inclusion of post-conflict country surveys in that review. It may be possible to cite more specific displaced population estimates.

Author Response: *Thank you for this feedback. Despite the growing number of refugees and asylum seekers worldwide there is a limited body of research, including recent systematic reviews, on the topic of refugee mental health. The authors agree that the systematic review conducted by Steel et al. 2009 is not completely aligned with the criteria from this current review as it included internally displaced and conflict-affected populations, however it is still an influential and highly cited paper in the field. We have now expanded this paragraph in*

the Introduction to offer a summary of the current research and the identified gaps in the literature. Please see the following changes in the Introduction, starting from line 99:

"...There is a however, a lack of estimates on the prevalence of mental illness in current global refugee populations as the literature has focused on either specific cultural groups or host nations. [8-11] *For instance, a recent systematic review of 8,176 Syrian refugees resettled in ten countries reported a prevalence of 43% for PTSD, 40% for depression and 26% for anxiety.* [12] *Not only is there is a lack of research which includes all global refugee populations, there is also a lack of research investigating the full breadth of mental illness as the literature has mainly focused on PTSD and depression.*

At line 101, page 4 the authors should clarify the number of systematic reviews examined and include citations for these.

Author Response: *Due to the update of the search, the number of systematic reviews examined has increased and this has been updated in the Method section at line 139. Since the number of systematic reviews that we examined is so large, 92 identified which resulted in an additional 37 articles to review, we feel that it is not practical to provide this information in the reference list. We would be happy to be guided by the editors and could include this list as part of the supplementary information.*

A difference between the Fazel et al study and this research which should be highlighted or corrected is that the authors have included LMI countries of first asylum studies of refugees and asylum seekers. The Fazel et al study was restricted to resettled refugees or asylum seekers in Western or HIC country settings.

Author Response: *Thank you for this feedback. We have made changes to the Introduction, Methods, and Discussion section to highlight the fact that the earlier systematic review focused on western countries and that this current review places no restrictions on resettlement countries. Please see the following sentences:*

Introduction, line 93:

“Fazel et al. 2005[6] conducted a systematic review and meta-analysis of refugees resettled in western countries, covering the period 1986-2004, and reported a prevalence of 9% for Post-Traumatic Stress Disorder (PTSD), 5% for Major Depressive Disorder, and 4% for Generalised Anxiety Disorder, based on studies reporting at least 200 participants.”

Methods, line 133:

“This review also placed no restrictions on resettlement countries.”

Discussion, line 357:

“The prevalence of PTSD and depression is higher than in the review by Fazel et al. [6] This could reflect the fact that this current systematic review included refugee populations from low- and middle-income countries…”

It is not clear that the search criteria are optimised to identify those displaced with the region as other terms are often applied. There are a number of studies amongst displaced populations that appear to have used structured diagnostic measures that may be relevant to stated inclusion criteria. I have included a list of studies that the authors should consider. If some of these do meet inclusion criteria then there may be problems with the search strategy applied.

Author Response: *Thank you for this feedback. We agree there can be many terms applied to study populations in the refugee field and we wanted to ensure that it would be a comprehensive search and not miss any important studies. The search strategy drew on the multidisciplinary clinical and research expertise of the authorship team (psychology, psychiatry, public health, refugee health, women’s health, systematic review and evidence synthesis) and the resulting 37 search terms and returned 21,842 records which were screened by two independent reviewers. Thank you for providing this list of studies for our consideration as it has given us the opportunity to cross check with our search results. All of these mentioned studies were found through our search, including two of the studies meeting inclusion criteria and being analysed in the meta-analysis. The full details of the screening of these studies are listed below.*

Banal, R., J. Thappa, et al. Psychiatric morbidity in adult Kashmiri migrants living in a migrant camp at Jammu.Indian J Psychiatry 52: 154-158; 2010).

Author Response: *This article was retrieved in the search and excluded based on full text review as the study sample included internally displaced populations.*

Amowitz LL, Heisler M, Iacopino V. A population-based assessment of women's mental health and attitudes toward women's human rights in Afghanistan. Journal of Women's Health. 2003;12(6):577-587.

Author Response: *This article was excluded based on full text review as the authors used a self-report questionnaire and cut off scores to determine depression diagnosis.*

Eytan A, Durieux-Paillard S, Whitaker-Clinch B, Loutan L, Bovier PA. Transcultural validity of a structured diagnostic interview to screen for major depression and posttraumatic stress disorder among refugees. Journal of Nervous & Mental Disease. 2007;195(9):723-728.

Author Response: *This article was excluded based on full text review as the MINI was administered by a nurse with no training in this instrument.*

Fenta H, Hyman I, Noh S. Determinants of depression among Ethiopian immigrants and refugees in Toronto. Journal of Nervous & Mental Disease. 2004;192(5):363-372.

Author Response: *This article was excluded based on full text review as the study included a mixed participant sample of immigrants and refugees and the resulting data were not disaggregated.*

Marshall GN, Schell TL, Elliott MN, Berthold SM, Chun C-A. Mental health of Cambodian refugees 2 decades after resettlement in the United States. JAMA. 2005;294(5):571-579.

Author Response: *This article was excluded by full text review. The authors of the study were contacted for further clarification regarding their methodology. The authors advised that the CIDI was administered, however the interviewers did not participate in the official WHO training, instead they were trained by staff who had attended official WHO training.*

Renner W, Salem I, Ottomeyer K. Cross-cultural validation of measures of traumatic symptoms in groups of asylum seekers from Chechnya, Afghanistan, and West Africa. Social Behavior and Personality. 2006;34(9):1101-1114.

Author Response: *This study met the inclusion criteria for this systematic review and was included in the meta-analysis.*

Renner W, Salem I. Post-traumatic stress in asylum seekers and refugees from Chechnya, Afghanistan, and West Africa: gender differences in symptomatology and coping. International Journal of Social Psychiatry. 2009;55(2):99-108.

Author Response: *This study met the criteria for inclusion in this systematic review. However, the data from this study were not extracted as it was the same dataset used in the Renner 2006 paper.*

Toscani L, Deroo LA, Eytan A, Gex-Fabry M, Avramovski V, Loutan L, Bovier P. Health status of returnees to Kosovo: do living conditions during asylum make a difference? Public Health. 2007;121(1):34-44.

Author Response: *This study was excluded based on full text review, as the sample assessed had been repatriated to their country of origin at the time of the mental health assessment.*

It is not clear what data was extracted by the authors - the manuscript lists sample size, publication year, and country or region of origin. Meta-analytic stratification suggest that other data was extracted such as, sex specific prevalence rates; duration of displacement, and living circumstance. Additional information should be provided on this and whether data on sex especially was extracted as a percentage distribution of extracted separately for males and females, the latter being preferable.

Author Response: *We have now added in further details regarding the exact data that were extracted in order to run the meta-analyses and subgroup analyses. The following sentences have been edited under the heading Data Analysis in the Methods section, please see line 166:*

"Using a fixed protocol two review authors (RB and MGH) independently extracted statistical data and study characteristics: host country, publication year, sample size, country or region of origin, sampling method, diagnostic tool and criteria, use of interpreter, age, proportion of female participants, visa status, duration of displacement, and prevalence of mental illness (numerator and denominator)."

It is a shame that information on the prior trauma and torture exposure given the importance of these as determinants of MH outcomes, although noted that this was not undertaken by Fazel but has been undertaken by subsequent reviews.

Author Response: *Thank you for this feedback. The authors agree that information on the types of trauma and torture experienced is of high importance particularly when investigating the prevalence of mental illness such as PTSD. In many of the studies, this information was not reported. We make a note of this lack of reported data in the discussion at line 337:*

> *"Although trauma type in relation to PTSD diagnosis was not adequately described in the studies…"*

Page 28, I agree a strength to limit to diagnostic measures, probably should also limit to multi-stage representative sampling. The study by Fazel especially suggested that study with a sample of over 200 may be the lowest number to reach stable population estimates.

Author Response: *The authors agree that this type of sampling is best practice and we would have liked to have restricted the inclusion criteria to only include studies which had incorporated a multi-stage representative sampling. However, when establishing our protocol, we were concerned that such a restriction in the field of refugee mental health would yield so few studies that the prevalence estimates could not have been made.*
