# Supplementary material for: The prevalence of mental illness in refugees and asylum seekers: A systematic review and meta-analysis
Source: PLoS Med. 2020 Sep 21;17(9):e1003337. doi: 10.1371/journal.pmed.1003337 (PMC7505461; doi:10.1371/journal.pmed.1003337)
Supplement: Attachment — Submitted filename: Response to Reviewers_3 July 2020.docx [file pmed.1003337.s009.docx]

**Requests from the editors:**

1. Reviewer 1, point #1: Please do address this point about native vs non-native interviewers raised by the reviewer, perhaps conducting a quick analysis to determine if there was any difference between native language and interpreter-assistance studies.

***Author Response:*** *Thank you for this feedback. We have now included in the manuscript an analysis to investigate any differences of reported prevalence between interpreter-assisted diagnostic assessments and those conducted in the native language. Figures 3, 5, and 7 have been updated to include this additional analysis. The results have been added to the Results section.*

*Please see the following results for the prevalence of PTSD, line 239:*

*“The PTSD prevalence for interpreter-assisted interviews was 35.75% (33.80-39.70) compared to 27.82% (26.40-29.30) for interviews conducted in the native language (p= <0.001).*

*Please see the following results for the prevalence of depression, line 264:*

*“The depression prevalence for interpreter-assisted interviews was 35.35% (95% CI 32.05-38.76) compared to 24.87% (95% CI 23.33-26.45) for interviews conducted in the native language (p = <0.0001).”*

*Please see the following results for the prevalence of anxiety, line 289:*

*“The use of an interpreter to conduct assessments had no significant impact on the reported prevalence of anxiety disorders (p = 0.34).”*

*Please see the following sentences added to the Discussion in response to these results, line 376:*

*“The prevalence of PTSD and depression appeared to be higher in studies that utilised interpreter-assisted diagnostic assessments. However, this was not the case for anxiety disorders, where there was no difference between the interpreter-assisted interviews and those conducted in the native language. This difference could be due to a number of factors, such as language fluency which plays an important role in the diagnosis of mental illness as the clinician relies heavily on the self-reported symptoms of the individual. However, further research is required to understand the differences in diagnosis rates between interpreter-assisted interviews and clinicians conducting the assessment in the native language and whether there are cultural and linguistic nuances which can impact on diagnostic rates that might only be accessible to native interviewers.”*

2. Response to Reviewers: Reviewer 2, point #2: In response to reviewer 2’s second point about studies which included populations from psychiatric clinics being excluded, I think please explicitly clarify in the text that you have included studies that recruited individuals from general refugee health/ primary health care clinics.

***Author Response:*** *We have included an additional statement in the Method section in order to explicitly clarify that this analysis included studies recruiting individuals from primary health care clinics.*

*Please see the following sentence in the Method section, line 157:*

*“Studies were excluded if:*

*-Participants were recruited from psychiatric or mental health clinics (to reduce selection bias). However, those studies which recruited participants from primary health care clinics were still included.”*

3. Response to Reviewers: Reviewer 2, point # 3: Please discuss ways in which the review's criteria could be a potential contributing factor to the heterogeneity observed.

***Author Response:*** *Thank you for this feedback. Upon revisiting the Discussion, we agree that our statements supporting the reviewer’s views were not clearly made and we have revised them. We believe that the main way in which the review’s criteria could have potentially contributed to the observed heterogeneity is through the few limits placed on characteristics of the study population such as country of origin or duration of displacement. In the Discussion, we have now clarified the following two statements in order to highlight this point more.*

*In the Discussion, please see line 347:*

*“With the aim of including all possible refugee populations that have been studied, this systematic review placed few restrictions on characteristics of refugee experiences (region of origin or resettlement, duration of displacement, etc.). As a result, the review’s criteria could in fact have been a contributing factor to the resulting substantial statistical heterogeneity.”*

*Continued in the Discussion, please see line 412:*

*“Some statistical heterogeneity is to be expected as a result of the review’s design which set no exclusion criteria for host country, country of origin, sex, or duration of displacement. We addressed this by using random effects models to calculate more conservative 95% confidence intervals.”*

4. Response to Reviewers: Reviewer 3, point #1: Please more clearly discuss that a limitation of some of the cultural/nation specific prevalence estimates (such as those that were cited) is that some estimates focus on internally displaced/conflict-affected populations and a global refugee comprehensive review is needed.

***Author Response:*** *Thank you for this feedback. Based on these comments, we have restructured the relevant paragraph of the Introduction in order to more clearly discuss the limitations of the current literature and highlight the need for this comprehensive systematic review.*

*Please see the following changes in the Introduction commencing line 101:*

*“As the literature has focused on either specific cultural groups, specific host nations, or has combined internally displaced populations with refugees and asylum seekers, there is a lack of estimates on the prevalence of mental illness in more representative refugee and asylum seeker populations [9-12]. There is also a lack of research investigating the full breadth of mental illness as the literature has mainly focused on PTSD and depression. Hence the need for a comprehensive, worldwide, systematic review to investigate mental illness in the current refugee populations.”*

*Please also see the changes to the last sentence of the Introduction, line 125:*

*“This systematic review aims to establish the current overall prevalence of mental illnesses in refugee and asylum seeker populations by summarising the current global body of evidence and overcoming some methodological limitations of individual studies.”*

5. Response to Reviewers: Reviewer 3: final point: Please discuss that restricting inclusion criteria to studies which used multi-stage representative sampling is a limitation (perhaps discuss within the discussion section), and please report in the manuscript how many of their studies would have been excluded based on this restriction.

***Author Response:*** *Thank you for this feedback. One of the difficulties in conducting research with refugee populations is recruitment and sampling. Restricting studies based on their sampling techniques was not possible as it would have severely limited the number of studies included in the review. As part of the risk of bias appraisal procedure, we examined each study in terms of their recruitment strategies in order to assign a rating. We discussed the findings of this process in the manuscript under the Risk of Bias heading in the Discussion as well as extracting this information for the Table of Included Studies.*

*We have also now added the following sentences to the Discussion, under the heading of Strengths and Limitations, to further discuss the points raised by the reviewer.*

*Please see line 418:*

*“There are many challenges to conducting research with refugee populations, one of which is sampling. Ideally, this review would have restricted the inclusion criteria to studies which incorporated multi-stage representative sampling. However, such a restriction in this field would have yielded so few studies that the prevalence estimates could not have been made. In fact, only two of the included studies in this review would have met this criterion.”*

6. Abstract: Line 48-49: Please clarify this sentence: “Random effects, based on inverse variance weights, were conducted.” Do you mean “random effects models” were conducted?

***Author Response:*** *Thank you for this feedback. We have now added the word ‘models’ to improve the clarity of this sentence.*

*Please see line 48 of the Abstract:*

*“Random effects models, based on inverse variance weights, were conducted. Subgroup analyses were performed for sex, sample size, displacement duration, visa status, country of origin, current residence, type of interview (interpreter-assisted or native language), and diagnostic measure.”*

7. Abstract: Please make some mention of the countries, or at least number of countries, represented by the review, such as described in your results (or similar): “Studies were undertaken in 15 countries with participants from four geographical regions: The Middle East (43%), Europe (29%), Asia (20%), Africa (5%).”

***Author Response:*** *Further details regarding the countries represented by the review have now been included in the Abstract.*

*Please see the following changes, commencing line 53:*

*“Twenty-six studies, which included one randomized controlled trial and 25 observational studies, provided results for 5,143 adult refugees and asylum seekers. Studies were undertaken across 15 countries: Australia (652 refugees), Austria (150), China (65), Germany (1104), Italy (297), Lebanon (646), Nepal (574), Norway (64), South Korea (200), Sweden (86), Switzerland (164), Turkey (238), Uganda (77), United Kingdom (420), and the United States of America (406).”*

8. Abstract: Methods and Findings: Please revise this sentence to clarify as follows: “The prevalence of post traumatic stress disorder (PTSD) was 31.46% (95% CI 24.43-38.5), the prevalence of depression was 31.5% (95% CI 22.64-40.38), the prevalence of anxiety disorders was 11% (95% CI 6.75-15.43), and the prevalence of psychosis was 1.51% (95% CI 0·63-2·40).” or similar.

***Author Feedback:*** *We have made the revision as requested to the Methods and Findings section in the Abstract.*

*Please see line 56:*

*“The prevalence of post-traumatic stress disorder (PTSD) was 31.46% (95% CI 24.43-38.5), the prevalence of depression was 31.5% (95% CI 22.64-40.38), the prevalence of anxiety disorders was 11% (95% CI 6.75-15.43), and the prevalence of psychosis was 1.51% (95% CI 0.63-2.40).”*

9. Abstract: Limitations statement: Thank you for including a statement of limitations, please revise to: “A limitation of the study is that substantial heterogeneity was present in the prevalence estimates of PTSD, depression, and anxiety, and limited covariates were reported in the included studies.”

***Author Feedback:*** *We have revised the limitation statement in the Abstract to include the suggested sentence.*

*It now reads, line 59:*

*“A limitation of the study is that substantial heterogeneity was present in the prevalence estimates of PTSD, depression, and anxiety, and limited covariates were reported in the included studies.”*

10. Abstract: Line 59: Please remove the subjective term “rigorous”

***Author Feedback:*** *The term ‘rigorous’ has been removed.*

11. Author summary: What do these findings mean? Line 77: Should this read “increased prevalence” in the first bullet point?

***Author Response:*** *In the author summary, we have updated the first bullet point under the heading – What do these findings mean? to now read:*

*“•	The increased prevalence of PTSD and depression appears to persist for many years after displacement.”*

12. Introduction: Line 94, and throughout manuscript where applicable: Please refer to low- or middle-income countries rather than "developing countries" or "the Global South". Please refer to high income countries rather than "developed" or "Western" countries.

***Author Feedback:*** *Thank you for this feedback. We have now revised this sentence in the introduction to read as follows (line 94):*

*“Fazel et al. 2005[6] conducted a systematic review and meta-analysis of refugees resettled in high-income countries…”*

13. Results Line 232-233: Please also provide 95% CIs for duration of displacement results.

***Author Response:*** *We have provided further details regarding the prevalence results and 95% CIs for the duration of displacement subgroup analysis of PTSD.*

*Please see the changes in the Results section, line 237:*

*“Duration of displacement had no significant impact on PTSD prevalence (p = 0.11). The prevalence of PTSD for those displaced less than four years was 30.17% (95% CI 28.24-32.14) compared to 33.14% (95% CI 29.99-36.41) for those displaced greater than four years.”*

14. Results Line 247-249: Please clarify “significantly lower” than which comparison group in the following sentence: “In the seven larger studies with 200 or more participants, depression prevalence was significantly lower, 20.65% (95% CI 18.88-22.51).” If statistical significance is meant, please include associated p values in addition to CIs.

***Author Response:*** *We have improved the clarity of this sentence.*

*Please see the following changes in the Results section, starting at line 260:*

*“In the seven larger studies with 200 or more participants, the reported depression prevalence was 20.65% (95% CI 18.88-22.51) which was significantly lower (p = <0.001) than the smaller studies, 32.89% (95% CI 30.06-35.82).”*

15. Results Line 249-250: Please also provide 95% CIs for duration of displacement results.

***Author Response:*** *We have improved this sentence.*

*Please see the following changes, starting at line 262:*

*“**Duration of displacement had no significant impact on depression prevalence (p = 0.17). The prevalence of PTSD for those displaced less than four years was 32.44% (95% CI 30.00-34.95) and 35.12% (95% CI 32.08-38.25) for those displaced greater than four years.”*

16. Results Line 271: Please also provide 95% CIs for the sample size and anxiety disorder analysis.

***Author Response:*** *Please see the following changes starting from line 287:*

*“Sample size had no significant impact on anxiety disorder prevalence (p = 0.21). The prevalence of anxiety disorders in the smaller studies (N <200) was 9.24% (95% CI 7.36-11.42) and in the larger studies (N≥200) the prevalence was 10.83% (9.50-12.27).*

17. Results Line 295-297: Please clarify that you meant “resulting in” and not “...and this was associated with fewer than expected…” in the following sentence: “Additionally, in one study only male psychologists conducted the diagnostic assessments, resulting in fewer than expected reports of sexual assault. [51]”

***Author Response:*** *We have edited this sentence for improved clarity.*

*Please see line 318:*

“*Additionally, in one study, only male psychologists conducted the diagnostic assessments, and this was associated with fewer than expected reports of sexual assault [51].*

18. Discussion: Line 314: Please indicate that you mean “anxiety prevalence” here.

***Author Response:*** *We have made the following changes to the sentence, starting at line 334:*

*“However, this was not the case for the prevalence of anxiety disorders which appeared to decrease with time.”*

19. Discussion: Line 344-346: Please revise this sentence to clarify: Although trauma type in relation to PTSD diagnosis was not adequately described in the studies, many studies reported on participants from countries such as the former Yugoslavia, Syria, and Iraq, areas with conflicts reported to have perpetrated systematic sexual violence.” or similar depending on your intended meaning here.

***Author Response:*** *Thank you for this feedback. We have made the suggested changes to this sentence.*

*Please see line 365:*

*“Although trauma type in relation to PTSD diagnosis was not adequately described in the studies, many of the studies included participants from countries such as the former Yugoslavia, Syria, and Iraq, areas with conflicts reported to have perpetrated systematic sexual violence [68].*

20. Discussion: Line 353: Please revise this sentence, without overreaching what can be concluded from the data- we suggest: “Our findings suggest that the prevalence of PTSD and depression persists for many years post-displacement…” Similarly, at line 363, we suggest beginning this sentence with “We found that…”

***Author Response:*** *We have made the suggested changes to both of these sentences.*

*Please see line 387 in the Discussion:*

*“Our findings suggest that the prevalence of PTSD and depression persists for many years post-displacement, suggesting ongoing suffering from mental illnesses in the post-migration environment.”*

*Please see line 397 in the Discussion:*

*“We found that the prevalence of PTSD and depression is higher than in the review by Fazel et al. [6]”*

21. References: In-text citations should be in regular text (not superscript), with numbers in brackets appearing before punctuation, like this: [1].

***Author Response:*** *We have made the changes to the in-text citations as requested. These changes have not been highlighted in the manuscript to maintain readability.*

22. Reference List: Please use the "Vancouver" style for reference formatting, and see our website for other reference guidelines: <https://journals.plos.org/plosmedicine/s/submission-guidelines#loc-references>

***Author Response:*** *We have revised the reference formatting in order to be in line with the Vancouver style requirements. These changes have not been highlighted in the manuscript to maintain readability.*

23. Supporting information: S1 Table, S3-S6 Eggers test results: Please provide titles and descriptive legends for each individual table and figure in the Supporting Information.

***Author Response:*** *We have provided titles and descriptive legends for the table and figures in the Supporting Information.*

Comments from the reviewers:

Reviewer #1: The revised version of the manuscript is much approved by the authors. The authors have spent significant effort addressing review amendments and suggestions. The responses were appropriate and the corresponding manuscript has reflected the suggested changes.

Two final additional points that I felt could potentially enhance this nice piece of work to consider:

1) There were thirteen studies conducting assessment in native language compared to thirteen with assistant from interpreters. I wonder if there was any influence on prevalence figures - which would have implications on the mode of assessment in native languages.

***Author Response:*** *Thank you for this feedback. We have now included an analysis to investigate any differences of reported prevalence between interpreter-assisted diagnostic assessments and those conducted in the native language. Figures 3, 5, and 7 have been updated to include this additional analysis. The additional results and discussion have been highlighted in the manuscript and also described above for reviewer 1, point 1.*

*Please see the following results for the prevalence of PTSD, line 239:*

*“The PTSD prevalence for interpreter-assisted interviews was 35.75% (33.80-39.70) compared to 27.82% (26.40-29.30) for interviews conducted in the native language (p= <0.001).”*

*Please see the following results for the prevalence of depression, line 264:*

*“The depression prevalence for interpreter-assisted interviews was 35.35% (95% CI 32.05-38.76) compared to 24.87% (95% CI 23.33-26.45) for interviews conducted in the native language (p = <0.0001).”*

*Please see the following results for the prevalence of anxiety, line 289:*

*“The use of an interpreter to conduct assessments had no significant impact on the reported prevalence of anxiety disorders (p = 0.34).”*

*Please see the following sentences added to the Discussion in response to these results, line 376:*

*“The prevalence of PTSD and depression appeared to be higher in studies that utilised interpreter-assisted diagnostic assessments. However, this was not the case for anxiety disorders, where there was no difference between the interpreter-assisted interviews and those conducted in the native language. This difference could be due to a number of factors, such as language fluency which plays an important role in the diagnosis of mental illness as the clinician relies heavily on the self-reported symptoms of the individual. However, further research is required to understand the differences in diagnosis rates between interpreter-assisted interviews and clinicians conducting the assessment in the native language and whether there are cultural and linguistic nuances which can impact on diagnostic rates that might only be accessible to native interviewers.”*

2) The authors picked up five diagnosis measures - were there enough studies in any one of those diagnostic measures to conduct a sub-group that were diagnostic measure specific. Again - if possible the results could indicate what particular measures may be contributing to the heterogeneity and whether there is any indication that certain diagnostic measures may be more sensitive towards picking up higher levels of the outcomes of interest.

***Author Response:*** *Thank you for this feedback. We have included an analysis to investigate any influence of diagnostic measure on the prevalence of post-traumatic stress disorder and depression. We were unable to conduct this analysis for anxiety as there were insufficient studies for each of the measures (Eleven studies provided data for anxiety disorder prevalence and seven of those used the M.I.N.I, two used the WHO-CIDI, one used the SCID, and one used the Adult Separation Anxiety Structured Interview (ASA-SI)). Figures 3, 5, and 7 have been updated to include the results of this analysis.*

*Please see the following results for the prevalence of PTSD, line 241:*

*“There was a statistically significant difference across diagnostic measures (p = <0.001) with the CAPS yielding a higher prevalence of PTSD (40.41%, 95% CI 36.20-44.70), followed by the WHO-CIDI (31.6%, 95% CI 28.20-35.20), the SCID (30.55%, 95%CI 28-33.20), and the M.I.N.I (25.8%, 95% CI 24-27.70).”*

*Please see the following results for the prevalence of depression, line 266:*

*“There was a statistically significant difference across diagnostic measures (p = <0.0001) with the SCID yielding a higher prevalence of depression (34.52%, 95% CI 31.74-37.39), followed by the M.I.N.I (30.55%, 95% CI 28.59-32.56), and the WHO-CIDI (5.02%, 95% CI 3.46-7.01).”*

*Please line the following results for the prevalence of anxiety, line 290:*

*“The subgroup analysis for diagnostic measure could be conducted due to insufficient studies for each measure.”*

*In the Discussion, please see line 383:*

*“Even though the different diagnostic measures are considered comparable in performance and diagnosis precision [71],* *our results suggest some differences.* *However, further investigation is required to understand potential differences in case identification between diagnostic measures, but was outside the scope of this review.”*
