# Supplementary material for: The prevalence of mental illness in refugees and asylum seekers: A systematic review and meta-analysis
Source: PLoS Med. 2020 Sep 21;17(9):e1003337. doi: 10.1371/journal.pmed.1003337 (PMC7505461; doi:10.1371/journal.pmed.1003337)
Supplement: Attachment — Submitted filename: Response to Reviewers_30 July 2020.docx [file pmed.1003337.s010.docx]

**Requests from Editors:**

1. Results Line 289-290: Please provide 95% CIs to accompany the p value reported here: “The use of an interpreter to conduct assessments had no significant impact on the reported prevalence of anxiety disorders (p = 0.34).”

***Author Response:*** *We have added the following sentence to now include the accompanying 95% confidence intervals. Please see line 292:*

*“The use of an interpreter to conduct assessments had no significant impact on the reported prevalence of anxiety disorders (p = 0.34). The prevalence of anxiety for interpreter assisted interviews was 9.70 (95% CI 7.50 – 12.30) and 11.04 (95% CI 9.76 – 12.40) for those interviews conducted in the native language.”*

2.Discussion: first paragraph, lines 334-335: Please revise this sentence: “However, this was not the case for the prevalence of anxiety disorders, which we found to be higher among those displaced less than four years.” or similar, to clarify.”

***Author Response:*** *We have revised this sentence to the following, please see line 340:*

*“However, this was not the case for the prevalence of anxiety disorders, which we found to be higher among those displaced less than four years.”*

3.Discussion: Line 340: Should this read “...less than the lifetime prevalence…”?

***Author Response:*** *We have revised this sentence to now read (see line 346):*

*“However, the prevalence of anxiety disorders (11%) and psychosis (1.5%) in refugees and asylum seekers appears to be less than the lifetime prevalence in general population samples: 16% [58] and 3% [59] respectively.”*

4.Discussion: Line 377: Please revise: “anxiety disorders, where there was no difference between…” to read “...anxiety disorders, where we did not find evidence for a difference between…”

***Author Response:*** *We have revised this sentence to the following, please see line 384:*

*“However, this was not the case for anxiety disorders, where we did not find evidence for a difference between the interpreter-assisted interviews and those conducted in the native language.”*

5.Discussion: Line 385-386: Please revise to clarify this sentence, we suggest: “Although beyond the scope of this review, further investigation is required to understand potential differences in case identification between diagnostic measures.” or similar.

***Author Response:*** *We have revised this sentence to the following, please see line 393:*

*“Although beyond the scope of this review, further investigation is required to understand potential differences in case identification between diagnostic measures.”*

6.Discussion: line 399-400: Please revise to clarify this sentence. We suggest: “In contrast, the results for anxiety disorders and psychosis are comparable with previously reported prevalence rates [6].” or similar.

***Author Response:*** *We have revised the sentence to the following, please see line 408:*

*“In contrast, the results for anxiety disorders and psychosis are comparable with previously reported prevalence rates [6].”*

7.Discussion: Line 416: Please change to “investigate potential sources of heterogeneity”

***Author Response:*** *We have revised the sentence to the following, please see line 424:*

*“The conventional method to investigate potential sources of heterogeneity is to conduct a meta-regression…”*

8.Figure 2: Please define the abbreviations for PTSD and CI in the legend.

***Author Response:*** *These abbreviations have been added to the legend of Figure 2. Please see line 241.*

9.Figure 3: Please provide an X axis label. Please spell out abbreviations for PTSD, CI, and for the diagnostic measures MINI, CAPS, WHO-CIDI, and SCID in the legend.

***Author Response:*** *We have added an X axis label and provided these details in the legend for Figure 3, please see figure file and line 245.*

10.Figure 4: Please define the abbreviation for CI in the legend.

***Author Response:*** *We have added this abbreviation to the legend for Figure 4. Please see line 272.*

11.Figure 5: Please spell out the abbreviations for CI, MINI, WHO-CIDI, and SCID. Please provide an X axis label.

***Author Response:*** *We have provided an x axis label and added the abbreviations to Figure 5. Please see figure file and line 277.*

12.Figure 6: Please spell out the abbreviation for CI in the legend.

***Author Response:*** *We have added this abbreviation to the legend for Figure 6. Please see line 300.*

13.Figure 7: Please spell out the abbreviation for CI in the legend, and please provide an X axis label.

***Author Response:*** *We have provided an x axis label and added the abbreviation for Figure 7. Please see figure file and line 306.*

14.Figure 8: Please spell out the abbreviation for CI in the legend.

***Author Response:*** *We have added this abbreviation to the legend of Figure 8. Please see line 314.*

15. Supporting information files S3-S6: Please provide legends for each file in which you describe all figure panels and spell out all abbreviations used within the figures.

***Author Response:*** *We have included all abbreviations used within the supporting information files S3-S6. The figure legends are included directly below the figure in the supporting information files.*

Comments from Reviewers:

Reviewer #1: I agree that the authors have done their part in adding the additional analyses. How interesting the results!

This implication being I think being that when designing these assessments - the method of assessment and diagnostic measure had a significant bearing on the proportion of cases being detected (the direction of association seemed to be consistently one way as well).

The limitation is that we don't of course know what is closer to the true answer without a known reference standard (arguably in this population this would be difficult to achieve) but it does raise the issues that careful consideration of the method of assessment and instrument of assessment in this population.

***Author Response:*** *Thank you for this feedback. Based on the above comments, we have added the following text – please see line 390:*

*“Even though the different diagnostic measures are considered comparable in performance and diagnosis precision [71], our results suggest some differences, which highlight the importance of careful consideration of the method and instrument used in the mental health assessments of refugee populations. Although beyond the scope of this review, further investigation is required to understand the potential differences in case identification between diagnostic measures.”*
